# A multicentric quality-control study of exercise Doppler echocardiography of the right heart and the pulmonary circulation. The RIGHT Heart International NETwork (RIGHT-NET)

**DOI:** 10.1186/s12947-021-00238-1

**Published:** 2021-01-20

**Authors:** Francesco Ferrara, Luna Gargani, Carla Contaldi, Gergely Agoston, Paola Argiento, William F. Armstrong, Francesco Bandera, Filippo Cademartiri, Rodolfo Citro, Antonio Cittadini, Rosangela Cocchia, Michele D’Alto, Antonello D’Andrea, Philipp Douschan, Stefano Ghio, Ekkehard Grünig, Marco Guazzi, Stefania Guida, Jaroslaw D. Kasprzak, Theodore John Kolias, Giuseppe Limongelli, Alberto Maria Marra, Matteo Mazzola, Ciro Mauro, Antonella Moreo, Francesco Pieri, Lorenza Pratali, Nicola Riccardo Pugliese, Mauro Raciti, Brigida Ranieri, Lawrence Rudski, Rajan Saggar, Andrea Salzano, Walter Serra, Anna Agnese Stanziola, Mani Vannan, Damien Voilliot, Olga Vriz, Karina Wierzbowska-Drabik, Robert Naeije, Eduardo Bossone, On behalf of the RIGHT Heart International NETwork (RIGHT-NET) Investigators

**Affiliations:** 1 Cardio-Thoracic-Vascular Department, University Hospital “San Giovanni di Dio e Ruggi d’Aragona”, Salerno, Italy; 2 Institute of Clinical Physiology, C.N.R, Pisa, Italy; 3 Department of Family Medicine, Faculty of Medicine, University of Szeged, Szeged, Hungary; 4 Department of Cardiology, University of Campania “Luigi Vanvitelli”, Naples, Italy; 5 Division of Cardiovascular Medicine, University of Michigan Medical Center, Ann Arbor, MI USA; 6 Heart Failure Unit and Cardiopulmonary Laboratory, IRCCS Policlinico San Donato University Hospital, Milan, Italy Heart Failure Unit, Cardiology University Department, IRCCS Policlinico San Donato, Milan, Italy; 7 Department for Biomedical Sciences for Health, University of Milano, Milan, Italy; 8 IRCCS SDN, Naples, Italy; 9 Department of Translational Medical Sciences, Federico II University, Naples, Italy; 10 Cardiology Division, A Cardarelli Hospital, Naples, Italy; 11 Division of Cardiology, Umberto I° Hospital Nocera Inferiore, Nocera Inferiore, Italy; 12 Medical University of Graz, Graz, Austria and Ludwig Boltzmann Institute for Lung Vascular Research, Graz, Austria; 13 Division of Cardiology, Fondazione IRCCS Policlinico San Matteo, Pavia, Italy; 14 Center of Pulmonary Hypertension, Thoraxklinik Heidelberg at Heidelberg University Hospital, Heidelberg, Germany; 15 I Dept. and Chair of Cardiology, Bieganski Hospital, Medical University of Lodz, Lodz, Poland; 16 A. De Gasperis Cardio Center, ASST Grande Ospedale Metropolitano Niguarda, Milan, Italy; 17 Cardiology Department, Careggi University Hospital, Florence, Italy; 18 Department of Clinical and Experimental Medicine, University of Pisa, Pisa, Italy; 19 Azrieli Heart Center and Center for Pulmonary Vascular Diseases, Jewish General Hospital, McGill University, Montreal, Quebec Canada; 20 Lung & Heart-Lung Transplant and Pulmonary Hypertension Programs David Geffen School of Medicine, UCLA, Los Angeles, USA; 21 Cardiology Division, University Hospital, Parma, Italy; 22 Department of Respiratory Diseases, Monaldi Hospital, University “Federico II”, Naples, Italy; 23 Piedmont Heart Institute, Marcus Heart Valve Center, Atlanta, USA; 24 Centre Hospitalier Lunéville, Service de Cardiologie, Lunéville, France; 25 Heart Centre, King Faisal Specialist Hospital and Research Centre, Riyadh, Saudi Arabia; 26 Free University of Brussels, Brussels, Belgium

**Keywords:** Right ventricle, Pulmonary hypertension, Exercise echocardiography

## Abstract

**Purpose:**

This study was a quality-control study of resting and exercise Doppler echocardiography (EDE) variables measured by 19 echocardiography laboratories with proven experience participating in the RIGHT Heart International NETwork.

**Methods:**

All participating investigators reported the requested variables from ten randomly selected exercise stress tests. Intraclass correlation coefficients (ICC) were calculated to evaluate the inter-observer agreement with the core laboratory. Inter-observer variability of resting and peak exercise tricuspid regurgitation velocity (TRV), right ventricular outflow tract acceleration time (RVOT Act), tricuspid annular plane systolic excursion (TAPSE), tissue Doppler tricuspid lateral annular systolic velocity (S’), right ventricular fractional area change (RV FAC), left ventricular outflow tract velocity time integral (LVOT VTI), mitral inflow pulsed wave Doppler velocity (E), diastolic mitral annular velocity by TDI (e’) and left ventricular ejection fraction (LVEF) were measured.

**Results:**

The accuracy of 19 investigators for all variables ranged from 99.7 to 100%. ICC was > 0.90 for all observers. Inter-observer variability for resting and exercise variables was for TRV = 3.8 to 2.4%, E = 5.7 to 8.3%, e’ = 6 to 6.5%, RVOT Act = 9.7 to 12, LVOT VTI = 7.4 to 9.6%, S’ = 2.9 to 2.9% and TAPSE = 5.3 to 8%. Moderate inter-observer variability was found for resting and peak exercise RV FAC (15 to 16%). LVEF revealed lower resting and peak exercise variability of 7.6 and 9%.

**Conclusions:**

When performed in expert centers EDE is a reproducible tool for the assessment of the right heart and the pulmonary circulation.

## Background

Exercise Doppler echocardiography (EDE) is standard practice for the evaluation of patients with coronary artery disease. The procedure is now increasingly used for the assessment of the right heart and the pulmonary circulation [1–5]. Echocardiography of the right heart mainly relies on estimates of right chambers dimensions (diameters/areas/volumes) and function (i.e. fractional area change, tricuspid annular plane systolic excursion (TAPSE) and of tissue Doppler–derived tricuspid lateral annular systolic velocity (S’) [2, 3]. Furthermore it estimates the components of the pulmonary vascular resistance equation, that is pulmonary artery pressure (PAP) from the maximum tricuspid regurgitation velocity (TRV), or the right ventricular outflow tract (RVOT) acceleration time (Act) of PA flow, wedged PAP from the ratio of transmitral flow E and mitral annulus e’ waves and cardiac output (CO) from the left ventricular outflow tract (LVOT) aortic flow. The RIGHT Heart International NETwork (RIGHT-NET) study was designed to comprehensively define limits of normal in right heart function and pulmonary circulation hemodynamics during EDE (diagnostic value) and to investigate the impact of abnormal responses on clinical outcome in individuals with overt or at risk of developing pulmonary hypertension (prognostic value) [6, 7]. The present report aims to provide a quality control analysis of left, right heart and pulmonary circulation resting and EDE measurements among 19 echocardiography laboratories with proven experience participating in the RIGHT-NET study [6, 7].

## Methods

**Fig. 1:**
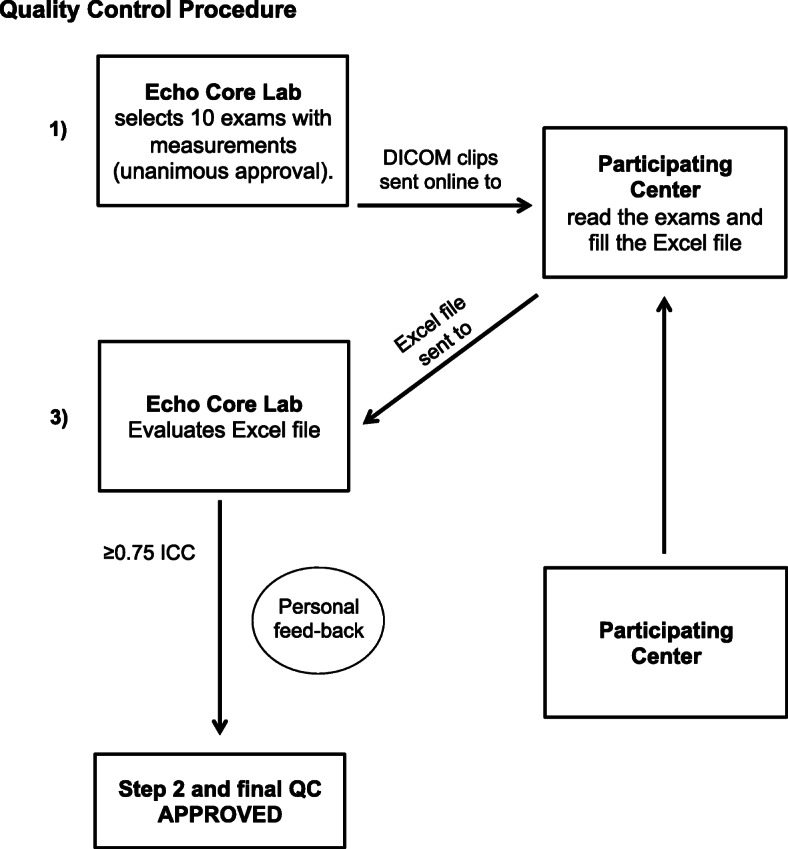
Quality control procedure

The echocardiography Core laboratory of the Institute of Clinical Physiology-CNR in Pisa (LG) coordinated the quality control procedure of all investigators at different centres participating in the RIGHT-NET study. Each center designated one operator that performed or reported at least 100 stress echocardiography studies per year. All readers were certified by national and/ or international societies. The quality control process was designed to be simple, reproducible and sustainable. The echocardiography Core laboratory issued a User Manual with a detailed description on how to measure each parameter, according to the most recent American and European Recommendations and Guidelines [8–11]. The User Manual was sent to all Participating Centers including the reference for transthoracic echocardiography assessment. All participating centers followed the recommended standard operational procedures in terms of data storage (data format, transfer procedure), and data processing (software used and measurement procedures). All operators performing and reading echocardiographic exams adhered to the quality control protocol. The echocardiography Core laboratory sent ten complete echocardiographic examinations in DICOM format through a safe file sharing platform (Fig. 1). All participating investigators were invited by email to join the platform, which was protected by user-specific passwords. The platform includes also detailed instructions on how to start the training and allows downloading and uploading of external files. Each reader was blinded to core laboratory measurements and to clinical history of the patients. All images and videos were completely anonymized to protect patients’ confidentiality’, in compliance with the EU’s General Data Protection Regulation 2018 [6].

### Reading sessions

The echocardiography core laboratory randomly selected 10 cases including healthy subjects and at least one group of patients with overt and/or at risk of pulmonary hypertension (PH), according to clinical classification of PH **(**Table 1**)** [12]. Echocardiographic examinations were performed with commercially available equipment on all subjects (Vivid E9, GE Healthcare, Milwaukee, WI, USA). Data were collected on patients undergoing EDE on a semi-recumbent cycle ergometer with an incremental workload of 25 W every 2 min up to the symptom-limited maximal tolerated workload including resting, 50 W, peak stress and recovery acquisition, as previously described [6]. All operators directly measured the requested parameters by uploading the same ten cases from the web platform to their echocardiography machine. The DICOM format enabled to perform assessment of variables in the respective centres. All operators were then asked to enter their measurements in a dedicated excel file, which was then sent to the Coordinating Center for analysis. Table 2 provides the list of the left and the right heart parameters measured by all operators. The gold standard value for each measurement was established by the values measured by the echocardiography Core laboratory, according to the recommendations for echocardiographic assessment of the left and right heart by the American Society of Echocardiography/European Association of Cardiovascular Imaging [8–11]**.**

**Table 1:** Demographic and clinical characteristics of 10 subjects included in quality control procedure

Variable	Value Mean ± SD
Age (years)	67.2 ± 11.3
Sex (male/female)	2/8
BSA (m^2^)	1.7 ± 0.2
BMI (Kg/m^2^)	25.2 ± 2.2
Systolic blood pressure (mmHg)	129 ± 24
Diastolic blood pressure (mmHg)	77 ± 12
Heart rate (bpm)	74 ± 11
**Diagnosis**	
Healthy subjects	1
vPAH	1
CTD	1
CHD	1
Patients with CAD risk factors	2
LHD	2
Lung disease	1
Post-PE	1

BSA, body surface area; BMI, Body Mass Index; CAD, coronary artery disease; CHD, congenital heart disease; CTD, connective tissue disease; LHD, left heart disease (coronary artery disease and heart failure); PAH, pulmonary arterial hypertension; Post-PE, post-pulmonary embolism; risk factors (hypertension, dyslipidaemia); SD, standard deviation. Data are expressed as number, mean ± SD

**Table 2:**
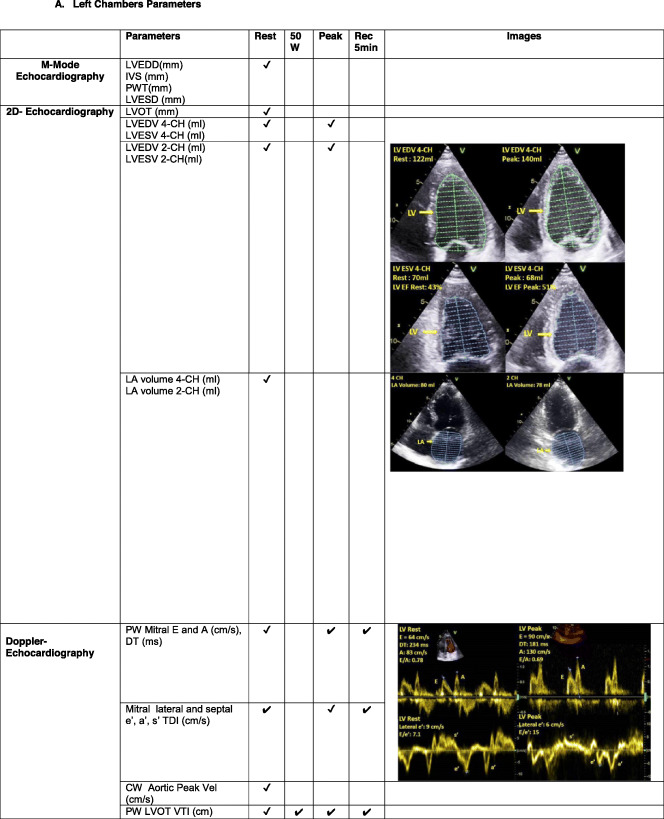

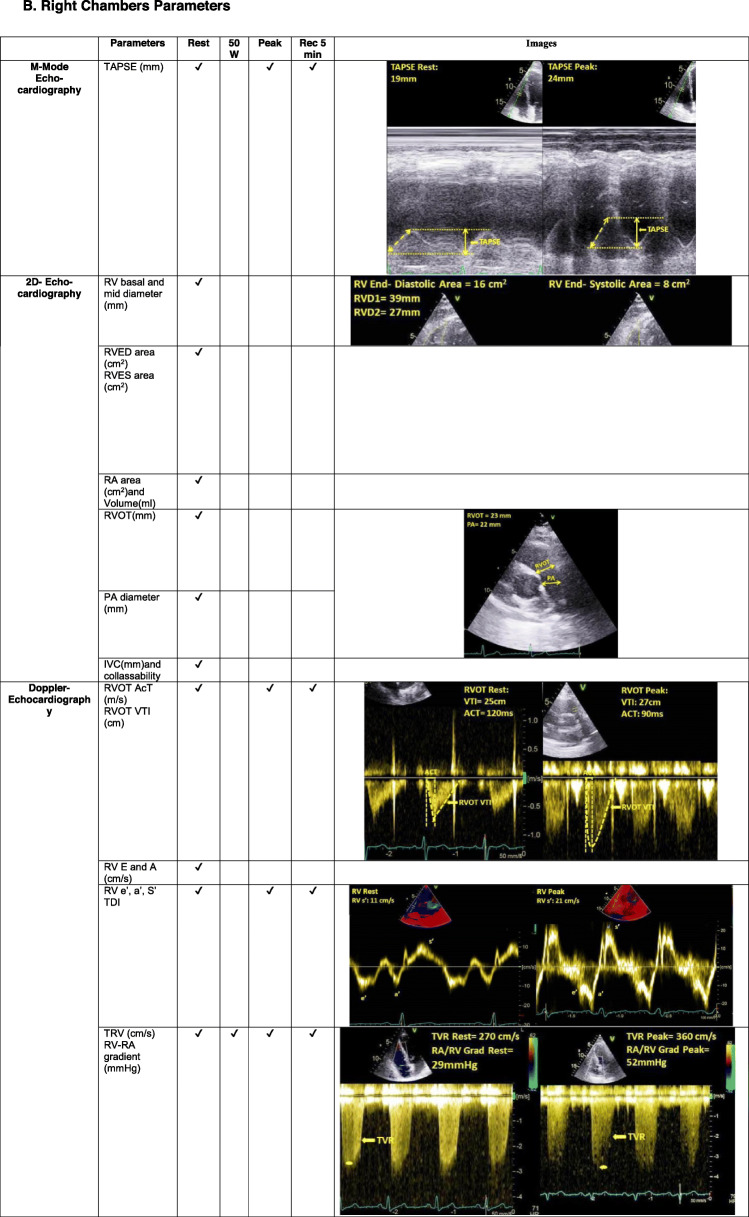
List of parameters measured in the quality control procedure, **A** Left Chambers Parameters. **B** Right Chambers Parameters

*AcT* acceleration time; *ED* end-diastolic; *ES* end-systolic; *IVC* inferior vena cava; *PA* pulmonary artery; *RA* right atrial; *Rec* recovery; *RV* right ventricular; *RVOT* right ventricular outflow tract; *TAPSE* tricuspid annular plane excursion; *TDI* tissue Doppler imaging; *TRV* trans-tricuspid valve regurgitation velocity; *VTI* velocity time integral

### Statistical analysis

Statistical analysis was performed using standard software (MedCalc version 14.8.1, MedCalc Software Ltd., Belgium; SPSS version 20.0, SPSS, Inc., Chicago, IL). Continuous variables were described by mean values ± standard deviation (SD). Normal distribution of the continuous values was assessed by the Kolmogorov-Smirnov test. Accuracy (in %) for each observer was estimated by comparison with the reference standard (core lab reading). Intra-class correlation coefficient (ICC) was calculated along with the 95% confidence interval, in order to quantify the reliability of measurement process. An ICC of > 0.8 indicated good agreement, ICC > 0.9 indicated excellent agreement with the core lab. Inter-observer variability among 19 observers were examined for resting and peak exercise TRV, RVOT Act, TAPSE, S’, right ventricular fractional area change (RV FAC), LVOT velocity time integral (VTI), mitral early inflow pulsed wave Doppler velocity (E), early diastolic mitral annular lateral and septal velocity by TDI (e’), left ventricular ejection fraction (LVEF). Data are presented as mean of the absolute and relative differences (in %) between measurements of all nineteen observers, and ICC for each single parameter was calculated along with the 95% confidence interval.

Intra-observer agreement was tested in 2 observers who volunteered to repeat the measurement session on 2 separate days and ICC was calculated.

## Results

**Fig. 2:**
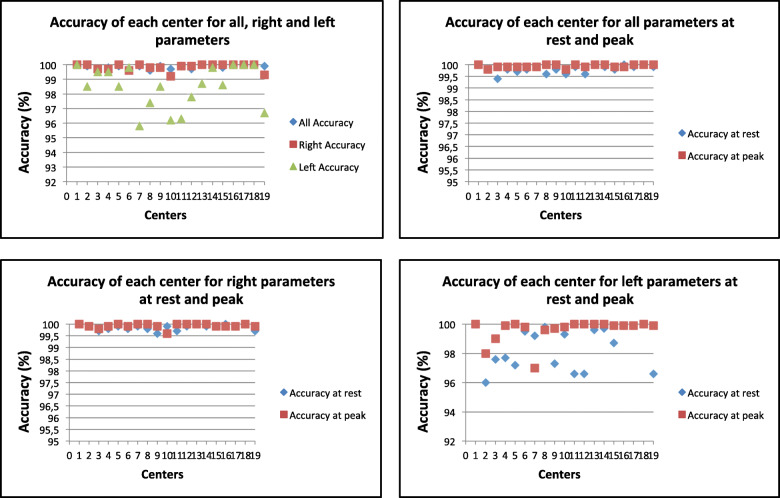
Accuracy of each center compared with the gold standard core lab for all, right and left parameters (**a**), for all parameters at rest and peak (**b**), for right paramaters at rest and at peak (**c**) and for left parameter at rest and at peak (**d**)

Nineteen observers completed all reading sessions. Figure 2 shows a summary of the accuracy (in %) of each center compared with the gold standard core lab for all parameters at rest and at peak of exercise. The average accuracy of 19 readers for all parameters was excellent in about 99.8% (range from 99.7 to 100%) (Table 3). ICC was > 0.9 for all observers. The average agreement of the 19 readers for all parameters was excellent (ICC = 0.98). Therefore there was no need to conduct personal feed-back and a second slot of measurements for anyone. Moreover the average agreement among readers remained excellent at rest and at peak exercise for all measurements (ICC = 0.98 and 0.99, respectively) (Table 4). Inter-observer variabilities among all observers for main exercise TTE measurements were reported in Table 5. Close inter-observer variabilities were found for both resting and peak exercise TRV (3.8 and 2.4%) (ICC = 0.97 and 0.98), RV S’ (2.9% for both) (ICC = 0.95 for both), E (5.7 and 8.3%) (ICC = 0.99 and 0.98) and e’ (6 and 6.5%) (ICC = 0.97 for both). Inter-observer variabilities of the RVOT Act and LVOT VTI were of 9.7% (ICC = 0.95) and 7.4% (ICC = 0.98) at rest, 12% (ICC = 0.92) and 9.6% (ICC = 0.97) at peak exercise, respectively. TAPSE showed less resting (5.3%) (ICC = 0.97) than peak exercise variability (8%) (ICC = 0.95). LVEF revealed lower resting and peak exercise mean relative differences of 7.6 and 9% (ICC = 0.99 and 0.98), respectively. Moderate inter-observer variability was found for resting and peak exercise RV FAC (15 and 16%, respectively) (ICC = 0.82 and 0.80) (Table 5).

**Table 3:** Accuracy, ICC and 95% Confidence Interval of each Center for all parameters

Centers	Accuracy (%)	*ICC*	*95% Confidence Interval*	
			*Lower bound*	Upper bound
*Center 1*	100	0.99	0.999	1.000
*Center 2*	99.9	0.99	0.991	0.997
*Center 3*	99.7	0.99	0.991	0.998
*Center 4*	99.8	0.99	0.992	0.998
*Center 5*	99.9	0.99	0.992	0.997
*Center 6*	99.7	0.99	0.993	0.998
*Center 7*	99.9	0.99	0.987	0.995
*Center 8*	99.6	0.99	0.988	0.996
*Center 9*	99.9	0.98	0.975	0.990
*Center 10*	99.7	0.98	0.975	0.996
*Center 11*	99.9	0.99	0.988	0.995
*Center 12*	99.7	0.99	0.990	0.997
*Center 13*	100	0.99	0.994	0.998
*Center 14*	100	0.99	0.999	1.000
*Center 15*	99.8	0.99	0.993	0.998
*Center 16*	100	0.99	0.999	1.000
*Center 17*	100	0.99	0.999	1.000
*Center 18*	100	0.99	0.999	1.000
*Center 19*	99.9	0.96	0.935	0.973

*ICC* = Intraclass Correlation Coefficient

p value < 0.0001 for each IC

**Table 4:** Accuracy, ICC and 95% Confidence Interval of each Center for all parameters at rest and peak

Centers	*Rest*				*Peak*			
	Accuracy (%)	*ICC*	*95% Confidence Interval*		Accuracy (%)	*ICC*	*95% Confidence Interval*	
			*Lower bound*	Upper bound			*Lower bound*	Upper bound
*Center 1*	100	0.99	0.999	1.000	100	0.99	0.999	1.000
*Center 2*	99.8	0.99	0.978	0.993	99.8	0.99	0.990	0.999
*Center 3*	99.4	0.99	0.982	0.997	99.9	0.99	0.994	0.999
*Center 4*	99.8	0.99	0.983	0.997	99.9	0.99	0.993	0.999
*Center 5*	99.7	0.99	0.984	0.995	99.9	0.99	0.994	0.999
*Center 6*	99.8	0.99	0.994	0.999	99.9	0.99	0.993	0.999
*Center 7*	99.9	0.99	0.991	0.997	99.9	0.99	0.993	0.999
*Center 8*	99.6	0.99	0.981	0.995	100	0.99	0.998	0.999
*Center 9*	99.8	0.98	0.972	0.991	100	0.99	0.979	0.996
*Center 10*	99.6	0.98	0.973	0.986	99.8	0.99	0.978	0.996
*Center 11*	99.9	0.99	0.990	0.997	100	0.99	0.975	0.996
*Center 12*	99.6	0.99	0.982	0.997	99.9	0.99	0.990	0.998
*Center 13*	100	0.99	0.995	0.999	100	0.99	0.996	0.999
*Center 14*	99.9	0.99	0.998	0.999	100	0.99	0.998	0.999
*Center 15*	99.8	0.99	0.990	0.997	99.9	0.99	0.991	0.999
*Center 16*	100	0.99	0.999	1.000	99.9	0.99	0.998	0.999
*Center 17*	99.9	0.99	0.998	0.999	100	0.99	0.998	0.999
*Center 18*	100	0.99	0.999	1.000	100	0.99	0.998	0.999
*Center 19*	99.9	0.99	0.975	0.992	100	0.99	0.992	0.999

*ICC* Intraclass Correlation Coefficient

p value < 0.0001 for each ICC

**Table 5:** Interobserver variability of main exercise Doppler echocardiographic measurements of all participating centers at rest and at peak exercise

	TRV (cm/s)	RVOT Act (msec)	TAPSE (mm)	RV S′ (cm/s)	RV FAC (%)	LVOT VTI (cm)	E (cm/s)	e’ (cm/s)	LV EF (%) MOD
**Rest**									
Mean	282 ± 22	128 ± 28	20.2 ± 2.2	12.2 ± 0.5	58 ± 8	20.5 ± 1.8	59.7 ± 5.3	12.0 ± 1.1	50 ± 4.6
Mean absolute difference	10.6 ± 10.8	13 ± 14	1.1 ± 1.2	0.4 ± 0.5	9.5 ± 7	1.6 ± 1.7	3.5 ± 4.2	0.7 ± 0.9	3.9 ± 3
Mean relative difference, %	3.8 ± 4	9.7 ± 9	5.3 ± 5.5	2.9 ± 3.9	15 ± 10	7.4 ± 7	5.7 ± 6.6	6.0 ± 6.9	7.6 ± 5.8
ICC	0.97	0.95	0.97	0.95	0.82	0.98	0.99	0.97	0.97
95% Confidence interval	0.92–0.99	0.93–0.99	0.92–0.99	0.88–0.98	0.75–0.91	0.95–0.99	0.97–0.99	0.93–0.99	0.93–0.99
**Peak exercise**									
Mean	322 ± 16	92 ± 16	24.2 ± 4.3	20.9 ± 1	59 ± 11	22.0 ± 2.8	82 ± 6.4	21 ± 1,6	57 ± 7
Mean absolute difference	7.7 ± 7.4	11 ± 11	1.5 ± 3	0.6 ± 0.8	9.2 ± 7.7	2.2 ± 2.4	7.3 ± 6.4	1.4 ± 1.2	5 ± 4
Mean relative difference, %	2.4 ± 2.3	12 ± 13	8 ± 14	2.9 ± 3.4	16 ± 14	9.6 ± 10	8.3 ± 7.0	6.5 ± 5.1	9 ± 7
ICC	0.98	0.92	0.95	0.95	0.80	0.97	0.98	0.97	0.96
95% Confidence interval	0.95–0.99	0.89–0.99	0.90–0.99	0.80–0.98	0.74–0.90	0.92–0.99	0.95–0.99	0.93–0.99	0.92–0.99

Legend: *Act* acceleration time; *CH* chamber; E, mitral early inflow velocity; *e’* early diastolic mitral annular lateral velocity; *EF* ejection fraction; *FAC* fractional area change; *ICC* intraclass correlation coefficient; *LVOT* left ventricular outflow tract; *MOD* biplane method of disks (modified Simpson’s rule); *RV* right ventricle; *RVOT* right ventricular outflow tract; S′, tissue Doppler–derived tricuspid lateral annular systolic velocity; *TAPSE* tricuspid annular plane systolic excursion; *TRV* tricuspid regurgitation velocity; *VTI* velocity time integral

The intra-observer quality control analysis revealed an excellent ICC of 0.97 (95% Confidence Interval: 0.96 to 0.99). All ICC > 0.95 remained excellent at rest and at peak exercise for all measurements, except for RV FAC (ICC = 0.85 and 0.82, respectively). Each ICC showed *p* value < 0.0001.

## Discussion

Before any acquisition of pooling echocardiographic data for research and clinical applications, a process of quality control and reading harmonization measurements should be undertaken [13–16]. The present results demonstrate that a rigorously designed protocol with a strong focus on quality assurance and certification can yield very strong ICC and limited variability among the 19 participant experienced centers to a large prospective EDE study of the right heart and the pulmonary circulation.

### Previous studies

The inter-observer variability during EDE right heart and pulmonary circulation studies may be not negligible. Few such studies have been previously reported and all were mono-centric [3, 17]. Argiento et al. reported in 124 healthy subjects (62 women and 62 men; age 37 ± 13 yrs) (single center study) an inter-observer variability for pulmonary artery systolic pressure (PASP) and cardiac output (CO) estimates of 1.9 and 4.9% at rest, and 7.9 and 13.9% at maximum exercise, respectively [18]. D’Alto et al. reported in 90 healthy subjects (45 male, mean age 39 ± 13 years) inter-observer variabilities between two readers at rest and peak exercise of 1.9 and 7.9% for PASP, 4.9 and 13.9% for stroke volume, 2.6 and 6.8% for TAPSE, and 5.4 and 8.7% for S′, respectively [19]. Kusunose et al. reported in a subgroup of 15 randomly selected subjects with isolated moderate to severe mitral regurgitation a close inter- and intra-observer variability for resting TAPSE (8.8%) and exercise TAPSE (9.5%) [20]. As these data remain limited, more validation appeared necessary for a multi-centric study like the RIGHT-NET.

### Uniqueness of the present study and clinical implications

To the best of our knowledge, this is the largest EDE multicenter study that comprehensively provides a detailed quality control analysis of both the right heart and the pulmonary circulation measurements. One major finding was that the accuracy and agreement were remarkably high among 19 experienced investigators, with no significant differences between resting and exercise measurements. These results provide a valid evidence of reliability of TRV, E/e’ ratio, LVOT VTI and LVEF during exercise. The inter-observer variability of RVOT Act was higher than that of TRV. RVOT Act measurements were collected during exercise, in keeping with a recent report advocating its combination with TRV for the assessment of the pulmonary pressures both at rest and during exercise [21]. The interest of this combination is that the feasibility rate of RVOT Act may be higher than that of TRV [22]. Furthermore our findings suggested that exercise TAPSE and S′ may be used as reproducible measures of the RV longitudinal systolic function. Larger resting and exercise variability of RV FAC may be caused by plane-dependency and reliance on a complex definition of the RV endocardial border [23].

### Study limitations

Few study limitations need to be discussed. First, the present study did not validate the echocardiographic measurements against invasive gold standard evaluation of the pulmonary circulation (PAP, wedged PAP and cardiac output), and right ventricular function (indices derived from pressure-volume loops). Second, accuracy and precision were defined by comparison only with the core laboratory measurements. In this regard, it was not logistically possible to repeat the echocardiographic examination of the same patient in each participating center. Third, the study results could have been potentially influenced by the quality images acquired only by the echocardiography core laboratory. For this reason we randomly selected 10 cases with different clinical conditions from a large database to avoid possible bias of selection of best images. Fourth, the number of patients studied was relatively small (*n* = 10). However, each of the 19 participating centers provided a total of 35 left and right heart echo-Doppler variables at rest, peak exercise and after 5 min of recovery.

## Conclusions

When protocols for acquisition and analysis are provided upfront and in experienced echocardiography laboratories EDE represents a reproducible tool to comprehensively assess the right heart and pulmonary circulation. This quality control study represents a solid bedrock for future RIGHT-NET studies, aiming to evaluate the diagnostic and prognostic role of EDE in the clinical settings of patients with cardiorespiratory diseases.

## Data Availability

The datasets analysed during the current study are available from the corresponding author on reasonable request.

## Appendix

**The RIGHT Heart International NETwork (RIGHT-NET) Investigators**

***Co-Principal Investigators:*** Eduardo Bossone (A Cardarelli Hospital, Naples, Italy), Luna Gargani (Institute of Clinical Physiology, CNR, Pisa, Italy), Robert Naeije (Free University of Brussels, Brussels, Belgium).

***Study Coordinator:*** Francesco Ferrara (Cava de’ Tirreni and Amalfi Coast Division of Cardiology, University Hospital, Salerno, Italy).

***Co-Investigators:*** William F. Armstrong, Theodore John Kolias (University of Michigan, Ann Arbor, USA); Eduardo Bossone, Rosangela Cocchia, Ciro Mauro, Chiara Sepe (A Cardarelli Hospital, Naples, Italy); Filippo Cademartiri, Brigida Ranieri, Andrea Salzano (IRCCS SDN, Diagnostic and Nuclear Research Institute, Naples, Italy); Francesco Capuano (Department of Industrial Engineering, Università di Napoli Federico II, Naples, Italy); Rodolfo Citro, Rossella Benvenga, Michele Bellino, Ilaria Radano (University Hospital of Salerno, Salerno, Italy); Antonio Cittadini, Alberto Marra, Roberta D’Assante, Salvatore Rega (Federico II University of Naples, Italy); Michele D’Alto, Paola Argiento (University of Campania “Luigi Vanvitelli”, Naples, Italy); Antonello D’Andrea (Umberto I° Hospital Nocera Inferiore, Italy); Francesco Ferrara, Carla Contaldi (Cava de’ Tirreni and Amalfi Coast Hospital, University Hospital of Salerno, Italy); Luna Gargani, Matteo Mazzola, Marco Raciti (Institute of Clinical Physiology, CNR, Pisa, Italy); Santo Dellegrottaglie (Ospedale Medico-Chirurgico Accreditato Villa dei Fiori, Acerra - Naples, Italy); Nicola De Luca, Francesco Rozza, Valentina Russo (Hypertension Research Center, University Federico II of Naples, Italy); Giovanni Di Salvo (University of Padova, Italy; Imperial College, London, UK); Stefano Ghio, Stefania Guida (I.R.C.C.S. Policlinico San Matteo, Pavia, Italy); Ekkerard Grunig, Christina A. Eichstaedt (Heidelberg University Hospital, Germany); Marco Guazzi, Francesco Bandera, Valentina Labate (IRCCS Policlinico San Donato, University of Milan, Milan, Italy); André La Gerche (Baker Heart and Diabetes Institute, Melbourne, Australia); Giuseppe Limongelli, Giuseppe Pacileo, Marina Verrengia (University of Campania “Luigi Vanvitelli”, Naples, Italy); Jaroslaw D. Kasprzak, Karina Wierzbowska-Drabik (Bieganski Hospital, Medical University of Lodz Poland); Gabor Kovacs, Philipp Douschan (Medical University of Graz, Graz, Austria); Antonella Moreo, Francesca Casadei, Benedetta De Chiara, (Niguarda Hospital, Milan, Italy); Robert Naeije (Free University of Brussels, Brussels, Belgium); Ellen Ostenfeld (Lund University, Skåne University Hospital, Sweden); Gianni Pedrizzetti (Department of Engineering and Architecture, University of Trieste); Francesco Pieri, Fabio Mori, Alberto Moggi-Pignone (Azienda Ospedaliero-Universitaria Careggi, Florence, Italy); Lorenza Pratali (Institute of Clinical Physiology, CNR, Pisa, Italy); Nicola Pugliese (Department of Clinical and Experimental Medicine, University of Pisa, Italy); Rajan Saggar (UCLA Medical Center,Los Angeles, USA); Rajeev Saggar (Banner University Medical Center, Phoenix, Arizona, USA); Christine Selton-Suty, Olivier Huttin, Clément Venner (University Hospital of Nancy, France); Walter Serra, Francesco Tafuni (University Hospital of Parma, Italy); Anna Stanziola, Maria Martino, Giovanna Caccavo (Department of Respiratory Disease, Federico II University, Monaldi Hospital, Naples, Italy); István Szabó (University of Medicine and Pharmacy of Târgu Mureș, Târgu Mureș, Romania); Albert Varga, Gergely Agoston, (University of Szeged, Szeged, Hungary); Darmien Voilliot (Centre Hospitalier Lunéville, France); Olga Vriz (Heart Centre, King Faisal Specialist Hospital and Research Centre, Riyadh, Saudi Arabia); Mani Vannan, Sara Mobasseri, Peter Flueckiger, Shizhen Liu (Piedmont Heart Institute, USA).
